# Genome Scans Reveal Species-Specific Selection in the Genus Lynx

**DOI:** 10.1093/gbe/evag086

**Published:** 2026-04-15

**Authors:** Lorena Lorenzo-Fernández, Enrico Bazzicalupo, Erin L Koen, Jan E Janecka, William J Murphy, Krzysztof Schmidt, José A Godoy

**Affiliations:** Department of Ecology and Evolution, Doñana Biological Station, CSIC, Seville, Spain; Department of Ecology and Evolution, Doñana Biological Station, CSIC, Seville, Spain; Wildlife Research and Monitoring Section, Ontario Ministry of Natural Resources, Peterborough, ON K9J 7B8, Canada; Department of Biological Sciences, Duquesne University, Pittsburgh, PA 15282, USA; Texas A&M University, Veterinary Integrative Biosciences, College Station, TX 77843, USA; Mammal Research Institute, Polish Academy of Sciences, Białowieża, Poland; Department of Ecology and Evolution, Doñana Biological Station, CSIC, Seville, Spain

**Keywords:** genome scan, selective sweep, lynx genus, positive selection, adaptation

## Abstract

Understanding the genetic basis of adaptation is essential for reconstructing evolutionary processes, and this can be accomplished particularly by studying closely related species occupying diverse ecological niches. In this study, we performed genome-wide scans for recent selective sweeps in the four extant species of the *Lynx* genus—*Lynx canadensis* (Canada lynx), *Lynx rufus* (bobcat), *Lynx lynx* (Eurasian lynx), and *Lynx pardinus* (Iberian lynx)—using a composite likelihood ratio test based on genotype frequency spectrum. Analyzing whole-genome sequences from 80 individuals, we identified species-specific selective sweeps and conducted functional enrichment analyses to explore biological processes under selection. Results revealed distinct adaptive mechanisms shaped by ecological specialization and demographic histories of different species. In Canada lynx, enriched functions include olfactory signaling and pigmentation-related processes; the Eurasian lynx showed signals related to cardiac and neural development; the Iberian lynx exhibited enrichment in immune-related pathways, potentially reflecting pathogen-mediated selection under strong genetic drift; and the bobcat displayed functional signals in reproductive and metabolic regulation. Our study revealed the species-specific nature of recent signatures of ecological differentiation in the genomes of closely related species of the genus Lynx, with minimal overlap, illustrating their diverse evolutionary trajectories and shedding light into the mechanism of adaptation among highly specialized carnivores.

Significance Closely related species provide an opportunity to explore how natural selection shapes genome-wide patterns of variation across contrasting ecological and demographic histories. By scanning whole genomes of all four extant lynx species, we identify recent, species-specific selective sweeps primarily associated with olfactory perception in Canada lynx, cardiac and neural function in Eurasian lynx, immune response in Iberian lynx, and reproductive and metabolic regulation in bobcat. These distinct adaptive signatures reveal that each species has followed unique evolutionary routes to ecological specialization, with limited evidence of shared selection signals despite some ecological overlap. Our results offer a comparative genomic perspective on adaptation in wild felids, highlighting how divergent ecological pressures and demographic legacies have shaped the evolutionary trajectories of the genus Lynx.

## Introduction

Examining the molecular basis of adaptive radiation can provide insight into the ecological and evolutionary processes that have shaped biological diversity (Edelsparre et al. 2024). Faced with rapidly changing biotic and abiotic conditions, understanding how species can and will adapt to future environmental conditions remains a fundamental question (Edelsparre et al. 2024; Urban et al. 2024). There is growing evidence that climate change is causing adaptive evolution in a variety of traits across multiple taxa (e.g. Prentice et al. 2017; Stonehouse et al. 2024; Forester et al. 2025), but there is still much to learn about the adaptive capacity and the molecular bases of adaptive responses in wild populations (Franks and Hoffmann 2012). Uncovering the processes that have shaped modern diversity can shed light on how species might cope with rapidly changing environmental conditions in the future (Edelsparre et al. 2024). Characterizing patterns of adaptive genetic variation in wild populations is an important first step (Bernatchez et al. 2024).

With the advent of genomics, genome scans have become a powerful tool for uncovering the genetic basis of evolutionary adaptation in wild populations (Vatsiou et al. 2016; Bernatchez et al. 2024). These statistical methods allow us to analyze variation along the genome to identify the genetic loci potentially affected by natural selection. Genome scans can detect the genomic footprint of a selective sweep, which is typically characterized by long, high-frequency haplotypes and reduced nucleotide diversity around the selected loci (Fay and Wu 2000; Kim and Stephan 2002; Sabeti et al. 2002; Voight et al. 2006). This occurs because alleles in the vicinity of the beneficial mutation hitchhike with the advantageous variant, rising in frequency faster than new variation can be introduced through mutation or recombination (Smith and Haigh 1974). Unlike traditional approaches that focus on putatively adaptive traits, genome scans allow the identification of candidate regions directly from genomic data, providing insights into recent adaptations (e.g. Johnston et al. 2011; Cho et al. 2013; Liu et al. 2014). These candidate regions are relevant not only for identifying targets of selection (e.g. Kardos et al. 2015; Makina et al. 2015; Giska et al. 2022; Joseph et al. 2023) but also for practical applications, such as delimiting species (Nosil et al. 2009), identifying conservation units (Funk et al. 2012; Prentice et al. 2019), and recognizing adaptively divergent populations (Després 2019).

**Fig. 1.**
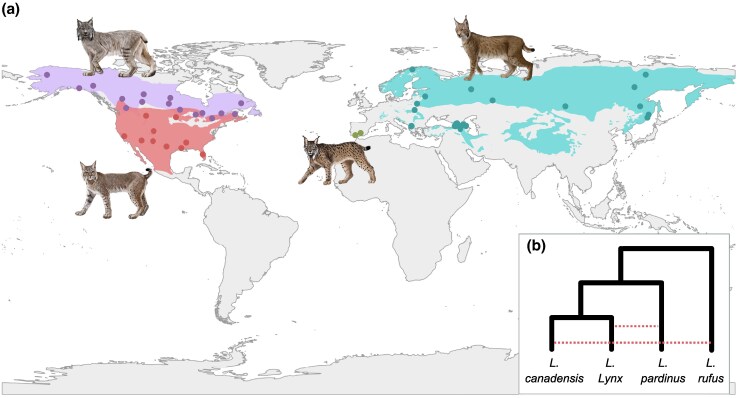
a) Distribution of Canada lynx (*Lynx canadensis*, purple), bobcat (*L. rufus*, red), Eurasian lynx (*L. lynx*, turquoise), and Iberian lynx (*L. pardinus*, green; note the small distribution in southwestern Europe). Symbols represent the approximate location of samples. When fine-resolution coordinates were unknown, we mapped the centroid of the country, state, or province. Six samples with unknown locations were not mapped. We obtained species distribution maps from International Union for the Conservation of Nature (2015, 2016), EU LIFE Lynxconnect (2024), Maine Department of Inland Fisheries and Wildlife (2016). Lynx illustrations © Paula Martin (@paulamartinart), used with permission. b) Species tree with dashed red lines indicating postspeciation admixture.

The lynx lineage diverged from other Felidae around 7 Mya (Murphy and Harris 2024), and diversified around 3 Mya (Johnson et al. 2006) in the four extant species: two distributed across the Nearctic—the bobcat (*Lynx rufus*) and the Canada lynx (*Lynx canadensis*)—and two found in the Palearctic—the Iberian lynx (*Lynx pardinus*) and the Eurasian lynx (*Lynx lynx*) (Fig. 1a). Although there's enough phylogenetic support for the monophyly of the genus (Walker 1991; Janczewski et al. 1995; Johnson et al. 1996; Johnson and O’Brien 1997), the topology of the tree relating the four extant lynx species and their biogeographic history remains debated. In contrast to previous studies (Johnson and O’Brien 1997; Li et al. 2016), recent phylogenomic approaches indicate alternative divergence patterns. Most autosomal genomic regions support an Iberian and Eurasian sister relationship, with their Most Recent Common Ancestor (MRCA) dated around 1 Mya. In contrast, low-recombination regions, which are less prone to introgression, reconstruct the Eurasian and Canada lynxes as sister species. This pattern suggests an older divergence of Iberian lynx followed by extensive introgression with the Eurasian lynx (Li et al. 2019) (Fig. 1b).

Lynxes are medium-sized wild cats characterized by their tufted ears, ruffed faces, and short tails, with species-specific differences in body size, coat pattern, tail markings, paw size, and facial ruff prominence. Like most other cats, they are solitary, territorial, crepuscular and use scent-marking for communication (Wilson and Mittermeier 2009; Hunter 2015). Lynxes have almost completely disjunct distributions, occupy different habitats and climatic zones, show distinct prey differences, and have contrasting demographic histories. Bobcat and Eurasian lynx are widely distributed, with the former occurring from Canada to southern Mexico and inhabiting temperate and boreal forests, and the latter ranging from the Atlantic in western Europe to the Pacific coast in Asia (Nowell and Jackson 1996). Both species are generalist and adaptable predators: bobcat diet consists mostly of lagomorphs, rodents and occasionally small ungulates whereas Eurasian lynx hunts preferentially medium-sized ungulates and lagomorphs (Newbury and Hodges 2018; Khorozyan and Heurich 2023). In contrast, Canada lynx and Iberian lynx are prey-specialist and thus their populations are prey density dependent. Canada lynx's habitat is confined to boreal forests in Canada, Alaska, and parts of the northern United States and relies heavily on the snowshoe hare (*Lepus americanus*) as their primary prey (Poole 2003; Lavoie et al. 2019), while Iberian lynx is restricted to Mediterranean forests and scrublands, and is highly dependent on the European rabbit (*Oryctolagus cuniculus*) (Palomares et al. 2001; Ferreras et al. 2004). In terms of body size, the Eurasian lynx stands out as the largest species, with adult males averaging 80 to 110 cm in head-body length and weighing 15 to 29 kg, whereas bobcat, Canada lynx, and Iberian lynx range from 65 to 105 cm and weigh 5 to 17 kg (Wilson and Mittermeier 2009). The distinct ecological niches occupied by each lynx species are likely the result of species-specific adaptations driven by their unique environmental pressures. These ecological differences, shaped by factors such as prey availability, climate and habitat, offer a valuable opportunity to investigate the genetic mechanisms underlying adaptation in these wild populations. However, studies explicitly targeting the genomic basis of adaptation in the genus *Lynx* are still remarkably few (Meröndun et al. 2019; Prentice et al. 2019, 2020; Broderick 2020; Harris et al. 2022; Bazzicalupo et al. 2023)

Here, we carried out genome scan analyses based on a composite likelihood ratio (CLR) test known as saltiLASSI (DeGiorgio and Szpiech 2022) to identify genomic regions with signatures of recent selective sweeps in each of the four extant lynx species. Our aim was to identify the genomic regions under selection in each species, the genes within these regions, and to test for enrichment of particular biological functions. By doing so, we seek to uncover key genetic adaptations underlying their ecological specialization. We also compare candidate regions across species to evaluate the extent and type of adaptations shared by two or more lynx species. A better knowledge of the lynx's adaptive landscapes can provide valuable insights into the evolutionary history of the genus and promote more effective conservation efforts by identifying the selective pressures that have been most relevant for their recent evolution and adaptation.

## Results

### Genomic Scans for Detecting Positive Selection

**Fig. 2.**
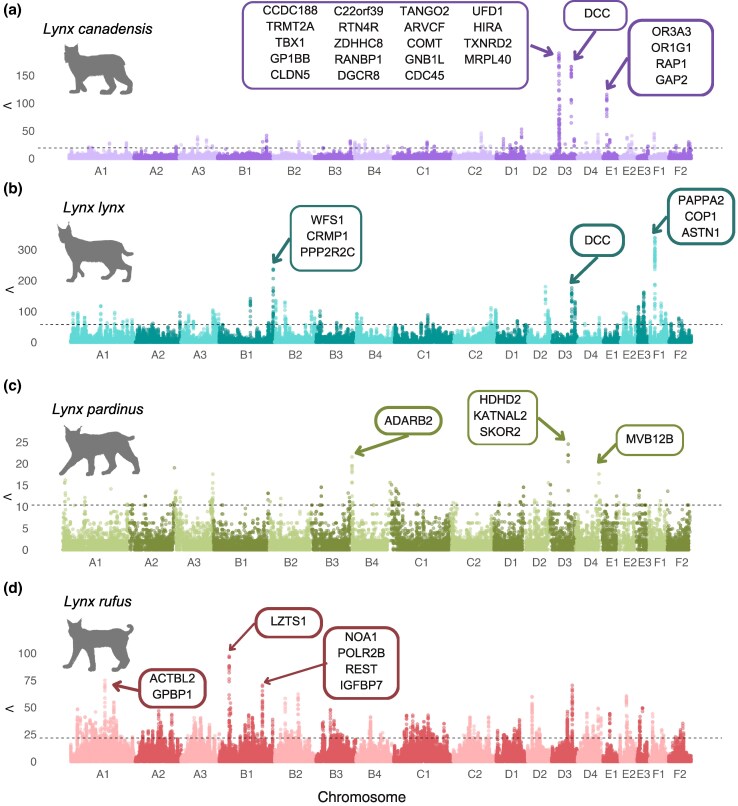
Genome-wide distribution of selection signals across the four lynx species using the saltiLASSI method. Manhattan plots show the distribution of selection scan statistics along the genome for *L. canadensis* (a), *L. lynx* (b), *L. pardinus* (c), and *L. rufus* (d). The horizontal dotted line indicates the 1% outlier threshold, representing the top candidate regions under positive selection. Labeled genes correspond to those located within the top three candidate regions with the highest statistic values in each species.

The final filtered datasets used for genomic scans of selection consisted of 1,486,086 SNPs for *L. canadensis,* 3,118,052 for *L. lynx,* 1,134,927 for *L. pardinus* and 8,848,061 for *L. rufus* (Table 1). The genome-wide distribution of selective sweep signals reveals distinct patterns across species, with strong, localized peaks in the Canada and Eurasian lynx, and more scattered signals in the bobcat and Iberian lynx (Fig. 2). Selection candidates were defined as those outlier windows with the 1% highest Λ values. After merging overlapping candidate windows, the number of candidate genomic regions was 55 for Canada lynx, 106 for Eurasian lynx, 74 for Iberian lynx and 259 for bobcat, with a median size of 165, 119, 158, and 88 kbp, respectively (Table 1, Table S2 to S5). Notably, the Canada lynx exhibited the largest candidate region, spanning more than 2 Mbps located on chromosome D4, albeit with a moderate statistic value, and overlapping with only one gene, *MLANA*, which is involved in melanogenesis. Given that the candidate region overlaps with the gene mainly in the expanded flanking regions, this signal may reflect regulatory variation upstream of the gene rather than changes in the coding sequence itself.

**Table 1:** Summary statistics from whole genome sequencing (WGS) and selection scans in the four lynx species

	*L. canadensis*	L. lynx	*L. pardinus*	*L. rufus*
*Samples*	19	32	11	18
*Mean coverage*	17X	21X	24X	21X
*Range coverage*	12 to 27X	9 to 35X	22 to 29X	17 to 26X
*N° SNPs*	1,486,086	3,118,052	1,134,927	8,848,061
*N° outlier windows*	286	617	176	1754
*N° candidate regions*	55	106	74	259
*Mean size (bp)*	222,607	157,685	182,732	121,892
*Median size (bp)*	165,162	119,038	158,128	87,800
*CDS*	158	160	165	330

### Strongest Selection Signals and Functional Characterization of Candidate Genes

**Fig. 3.**
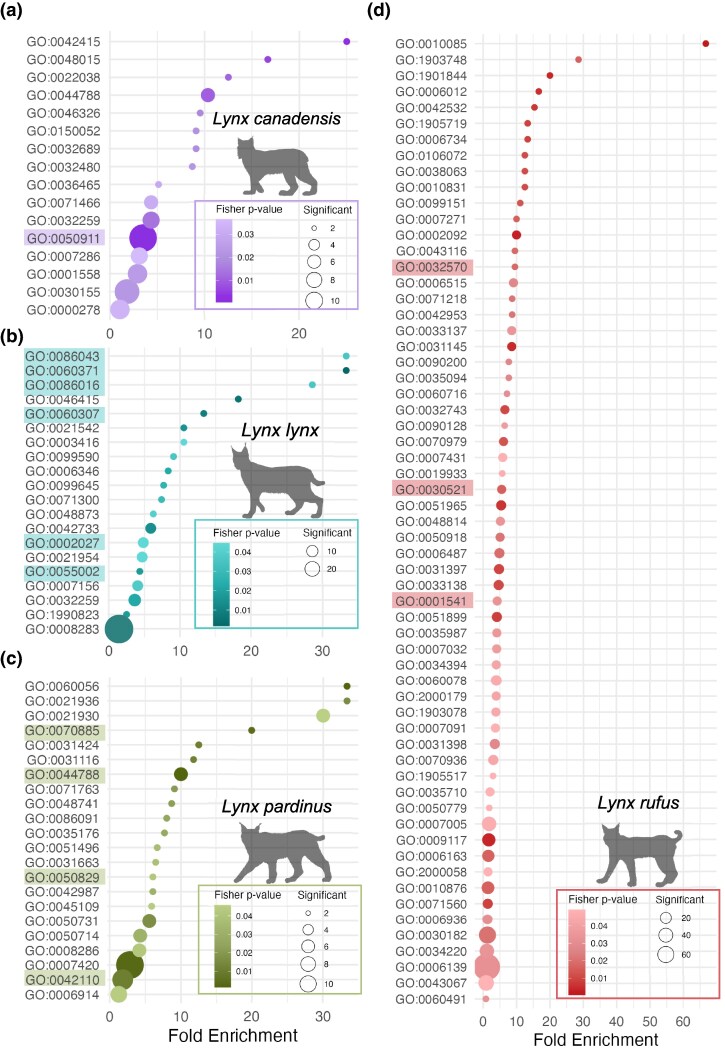
Functional enrichment and overlap of candidate genes under selection in *lynx* species. a–d) Gene Ontology (GO) term fold enrichment (ratio of observed to expected gene counts) for genes located in candidate regions under selection in *L. canadensis* (a), *L. lynx* (b), *L. pardinus* (c), and *L. rufus* (d). Dot size represents the number of candidate genes per GO term; color intensity reflects the statistical significance (Fisher's exact test *P*-value). GO terms description available in Tables S6 to S9. Highlighted GO terms correspond to functional categories emphasized in the discussion, sensory perception of smell in *L.canadensis*, cardiac function in *L. lynx*, antiviral and immune regulation in *L.pardinus* and reproductive regulation and gametogenesis in *L. rufus.*

In the Canada lynx, we observed prominent peaks on chromosomes D3, and E1, encompassing genes involved in neurodevelopment and signaling (*RTN4R, COMT, GNB1L, ARVCF, TBX1, DCC*), gene expression regulation (*DGCR8, RANBP1*, and *HIRA*), mitochondrial function (*TXNRD2, MRPL40*) and olfactory signaling (e.g. OR*3A3, OR1G1)* (Fig. 2a). From the candidate gene set (*n* = 158), we identified 16 significantly overrepresented GO terms (*P*-value < 0.05; Fig. 3a, Table S6), predominantly related to sensory perception of smell (GO:0050911), host–virus interaction and xenobiotic response pathways (GO:0044788, GO:0032689, GO:0032480, GO:0071466), development of neural connections (GO:0022038), intracellular signaling pathways (GO:0048015), norepinephrine biosynthetic process (GO:0042415), and metabolic regulation involving glucose uptake (GO:0046326).

The Eurasian lynx genome scan revealed significant peaks on chromosomes B1, D2, D3 and F1, with genes involved in neural development and function (*ASTN1, CRMP1, PPP2R2C, DCC*), growth regulation (*PAPPA2*) and stress response (*WFS1, COP1*) (Fig. 2b). From the candidate gene set (*n* = 160) we identified 20 enriched GO terms (*P*-value < 0.05; Fig. 3b, Table S7). The strongest signals were observed in terms related to cardiac function (GO:0086043, GO:0060371, GO:0060307, GO:0086016, GO:0002027, GO:0055002). Additionally, enriched terms related to neural development and synaptic signaling were identified (GO:0021542, GO:0021954, GO:0099590, GO:0099645). We also found terms associated with morphogenesis (GO:0042733, GO:0003416), as well as terms related to urate metabolism (GO:0046415).

In the Iberian lynx, the signal was generally weak. Nevertheless, we found notable peaks in regions overlapping genes involved in neural development (*ADARB2, SKOR2*) and metabolic regulation (*HDHD2*) (Fig. 2c). From the candidate gene set (*n* = 165), 22 significantly enriched GO terms (*P*-value < 0.05; Fig. 3c, Table S8) were identified, primarily related to antiviral and immune regulation (GO:0044788, GO:0042110, GO:0070885, GO:0050829) and neural development (GO:0021936, GO:0021930, GO:0007420). We also found several terms broadly involving muscular tissue development and structural regulation (GO:0031116, GO:0031424, GO:0048741, GO:0086091) and mammary gland regression after lactation (GO:0060056).

Finally, in the bobcat, we observed the strongest signals on chromosomes A1, B1 and D3, with genes associated with cytoskeletal integrity (*ACTBL2, LZTS1*), transcriptional control (*GPBP1, POLR2B, REST*), and stress resilience through mitochondrial function and growth regulation (*NOA1, IGFBP7*) (Fig. 2d). From the bobcat candidate gene set (*n* = 330), we identified 61 enriched GO terms (*P* < 0.05; Fig. 3d, Table S9), particularly related to protein homeostasis (GO:0006515, GO:0071218, GO:0070979, GO:0031397, GO:0033138), protein transport and localization (GO:0034394, GO:1903078, GO:1903748, GO:1905719, GO:0042953), and post-translational modifications (GO:0006487). We also found enriched terms involved in neuronal development and signaling, especially synapse assembly, maturation, and neurotransmission (GO:0051965, GO:0099151, GO:0090128, GO:0007271, GO:0051965, GO:0048814, GO:0060078). We also found terms related to reproductive regulation and gametogenesis (GO:0032570, GO:0030521, GO:0001541). Finally, metabolic processes were predominantly associated with carbohydrate and energy metabolism (GO:0006012, GO:0006734).

Protein–protein interaction networks inferred using the STRING database revealed that candidate genes were significantly more interconnected than expected by chance in all four lynx species (PPI enrichment *P* < 1.0 × 10^−16^ for Canada and Iberian lynx; *P* = 1.21 × 10^−5^ for Eurasian lynx and *P* = 7.05 × 10^−7^ for bobcat) (Table S10). Clustering analysis further identified significantly enriched protein groups in Canada lynx (FDR = 1.6 × 10^−3^ and 5.2 × 10^−3^), associated with olfactory learning, and in Iberian lynx (FDR = 1.45 × 10^−5^), associated with modulation by host of viral transcription (Table S11).

### Sharing of Candidate Genes Among Species

**Fig. 4.**
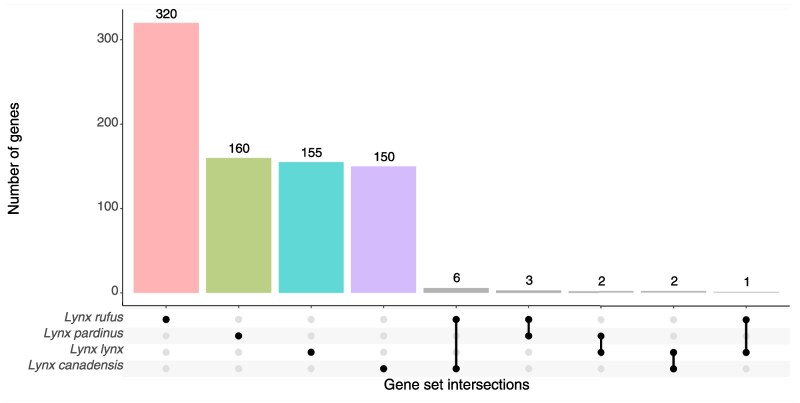
UpSet plot showing the number of shared and unique genes under selection among four species of *lynxes*.

To assess the presence of shared selection signals, we analyzed the overlap of candidate genes between pairs of lynx species. Because differences in demographic history and levels of genetic diversity among species may affect the power to detect selective sweeps, cross-species comparisons should therefore be interpreted cautiously. Overall, we found very little overlap in the positively selected genes across lynx species (Table S12). While no overlapping gene was observed between the Iberian and Canada lynx, minimal overlap was found between the other pairwise comparisons (Fig. 4). We identified six genes shared between bobcat and Canada lynx, including those involved in mitochondrial dynamics (*MYO19, MRM1*), protein and lipid metabolism (*PIGW, DHRS11*), and immune response (*GGNBP2*). Additionally, three candidate genes were candidate both in bobcat and Iberian lynx, and these are involved in embryonic development (*NODAL*), cellular growth (*EIF4EBP2*) and angiogenesis and vascular remodeling (*PALD1*). Eurasian lynx shares two candidate genes involved in neuronal signaling and regulation of neuronal excitability (*STK32B, DPP6*) with Iberian lynx, and only one with bobcat, which is associated with lipid metabolism (*LDAH*). Finally, we identified *DCC* and *CDH7* as candidate genes in both the Eurasian lynx and Canada lynx, associated with neuronal development and cell adhesion. Both the Canada lynx and Eurasian lynx share one of the highest conspicuous signals in a region that includes the *DCC* gene.

## Discussion

In this study, we identified candidate genomic regions under selection in the four extant lynx species, which contained genes involved in several biological processes of ecological relevance. These selection signals were not generally shared across species, which illustrate their different evolutionary trajectories and possible mechanisms of speciation within the genus *Lynx*, comprising ecologically highly diverse carnivores.

### Species-Specific Selective Pressures in *the genus Lynx*

Our findings reveal that recent selective sweeps in lynxes are clearly species-specific, driven by distinct evolutionary pressures that likely reflect each species’ unique ecological niche and selective forces. The variability in adaptive signals among closely related species with recent divergence underscores the evolutionary plasticity that enables felids to rapidly adapt to diverse environmental challenges (Johnson et al. 2006; Figueiró et al. 2017)

In Canada lynx, our overrepresentation results suggest significant selective pressure on olfactory perception, particularly in olfactory receptor (OR) genes. The OR repertoire is encoded by a multigene family responsible for detecting chemosensory signals and is likely the largest multigene family in vertebrates (Niimura 2012). Broad studies on OR evolution in mammals and vertebrates have found species-specific duplications in OR genes correlating with ecological adaptation, which suggests that the OR repertoire evolves in response to environmental demands (Hayden et al. 2010; Niimura 2012; Hughes et al. 2018). In felids, selection on OR gene families has been documented for several species, including domestic cat (*Felis catus*), cheetah (*Acinonyx jubatus*), fishing cat *(Prionailurus viverrinus*) and *Panthera* species (Montague et al. 2014; Dobrynin et al. 2015; Figueiró et al. 2017; Zhou et al. 2021; Bredemeyer et al. 2023). In addition to the role of smell perception in intra-specific communication, there is evidence that predators rely on olfaction to locate prey, particularly in environments where other sensory modalities, like vision, may be less effective (Hughes et al. 2010). In particular, Studd et al. (2022) showed that Canada lynx significantly increase feeding events on windy days, suggesting that olfaction is key for efficient hunting in boreal forests, where vision is limited. Notably, we also identified a remarkably long candidate region on D4, which includes only a single gene. This gene, *MLANA*, is primarily expressed in melanocytes and regulates the maturation of melanosomes, essential for melanin production in hair follicles. While the gene primarily responsible for the dilution color phenotype in domestic cats has been identified as *MLPH* (Ishida et al. 2006), mutations in *MLANA* have been shown to cause changes in pigmentation in other species, such as the dilution of plumage in Chinese yellow quail (*Coturnix japonica*) (Zhang et al. 2025) and lighter coats in knockout mice (Aydin et al. 2012). While the reason for such an extended region under selection (> 2 Mbps) requires further investigation, the role of *MLANA* in melanogenesis could be related to the observed coat color variation in Canada lynx. This variation includes individuals showing a bluish-gray or diluted pelage, especially during summer (Jones 1923; Schwarz 1938; Hunter 2015; Lavoie et al. 2019) lacking the distinct dark spotting patterns characteristic of other lynx species, although a rare case of melanism has also been reported (Jung 2023). Studies of coat color variation in Felidae are focused primarily on melanism, involving genes like *ASIP* and *MC1R* (Eizirik et al. 2003), but no studies have addressed the genetic basis of other coat phenotypes in Canada lynx. The variation in coat color may provide important adaptive advantages, such as thermoregulation or camouflage in the snowy, forested environments of their habitat.

In the Eurasian lynx, we identified several GO terms related to the regulation of cardiac activity, suggesting potential adaptations that support the energy demands of large home ranges or intense hunting effort required to capture larger prey (Hunter 2015). While there is evidence of cardiovascular gene adaptations to hypoxia in other species (Rong et al. 2012; Storz 2021; Zhao et al. 2024), the role of cardiac adaptations in enabling hunting efficiency or supporting extensive home ranges remains largely unexplored. However, a study on pumas (*Puma concolor*) showed that their energetic expenditures increase with prey size (Williams et al. 2014). Therefore, felids adapted to kill large prey like Eurasian lynx (Khorozyan and Heurich 2023) may have evolved anatomical features enabling them to cope with the associated challenges. Anatomical studies have revealed unique cardiac features in the Eurasian lynx when compared to other felids (Kareinen et al. 2020). Although its overall cardiac structure is consistent with that of other felids, the species exhibits distinctive thin ventricular bands with a fibromuscular pattern. This could at least partly explain the capability of this relatively small felid (17 to 22 kg) to subdue prey that is equal to or 3 to 4 times its own weight (Zbyryt et al. 2018). Although the adaptive significance of these anatomical differences remains unproven, our findings collectively highlight the need for further research to elucidate the evolutionary, ecological, and physiological implications of cardiac structure and metabolism in the Eurasian lynx.

For the Iberian lynx, we found a significant overrepresentation of immune-related terms. This species is currently recovering from severe demographic bottlenecks that reduced genetic diversity and likely increased vulnerability to pathogens. Indeed, similar patterns have been observed in other mammals like the African cheetah and Tasmanian devil (*Sarcophilus harrisii)*, where reduced diversity in immune response genes was suggested to compromise their ability to combat emerging diseases (Dobrynin et al. 2015; Peel and Belov 2018). In addition, genomic analyses have shown that the Iberian lynx has historically maintained low effective population size over relatively long evolutionary timescales (Abascal et al. 2016), and that its species-wide genetic diversity has remained exceptionally low, only recently increasing due to introgression from Eurasian lynx (Lucena-Perez et al. 2024). However, despite the severe genomic erosion, Marmesat et al. (2017) found substantial functional variation at the major histocompatibility complex (MHC) in the Iberian lynx, although evidence for positive selection in this gene family was limited. This contrasts with findings in other felids, such as the cheetah, Bengal tiger (*Panthera tigris*) and leopard (*Panthera pardus*), where positive selection on MHC genes has been reported (Pokorny et al. 2010; Castro-Prieto et al. 2011; Formenti et al. 2022). Altogether, the observed signal of immune adaptation in the Iberian lynx suggests strong and persistent pathogen-mediated selection, which, under prolonged demographic constraint, could have shaped immune gene evolution through compensatory adaptations. In such drift-dominated populations, only strong selection pressures—such as those from pathogens—are likely to overcome genetic drift, maintaining or favoring functionally relevant alleles essential for survival (Kimura 1983; Crow 1986).

In the bobcat, the enrichment results revealed, among others, significant overrepresentation of reproduction-related genes. Notably, the bobcat is the only lynx species with a more typical felid reproductive cycle, characterized by recurrent estrus cycles, and consequently ovulation, until successful fertilization (Jewgenow et al. 2014; Antonevich and Naidenko 2023). This contrasts with the other lynx species, which are seasonal breeders, exhibiting a single estrus per year to synchronize cub births with optimal environmental conditions. The maintenance of this feline-like reproductive strategy in bobcats may reflect an ancestral adaptation to their less seasonally-variable habitats, where resource availability remains relatively stable throughout the year (Delibes et al. 1997). This strategy may provide a fitness advantage by increasing reproductive output when resources are available. Although speculative, our findings suggest bobcat reproduction is adapted to its particular ecology, a hypothesis that should be further investigated to elucidate the molecular and ecological mechanisms underlying these adaptations and how they contribute to the reproductive success and population dynamics of bobcats.

Broderick (2020) used F_ST_ scans to identify ecologically associated selective sweeps within bobcat populations and in comparison with Canada lynx. Although the overall overlap of their results with ours is scarce, it includes several genes—*ME1, MTTP, WAPL, ADGRB3, and CADM2*—primarily involved in lipid metabolism, energy homeostasis, and neurodevelopment and behavior. Our findings also align with broader felid studies: we detected signals of positive selection in *UBE3A, PDE3A,* and *SSTR4* in bobcat, loci previously highlighted in big-cat genomes for their roles in pigmentation, reproductive physiology, and craniofacial development (Figueiró et al. 2017). Classical coat-pattern regulators first described in domestic cats likewise emerged as outliers—*EDAR* and *ASIP* in bobcat, and *EDNRB* in Eurasian lynx—mirroring developmental studies linking these genes to tabby patterning and white spotting (Kaelin et al. 2021). Additionally, we identified *EPAS1* and several FGF family genes within candidate regions in Canada lynx, previously implicated in hypoxia response and limb development, respectively (Cho et al. 2013; Wei et al. 2021). Taken together, this growing body of evidence suggests that, albeit limited, selection in the genus *Lynx* recurrently targets developmental, metabolic, and sensory pathways, often in parallel with other felid lineages.

### Limited Evidence of Parallel Evolution Between Lynx Species

We found no overlap in selection signals between the Iberian lynx and Canada lynx, and only minimal overlap between other species pairs. Because divergence among lynx species largely predates the temporal window detectable by genome-wide selection scans, any shared signals would most plausibly reflect independent (parallel) selection rather than retention of ancestral selective events. Based on ecological similarities among species, we expected to find some overlap in candidate genes under selection. In particular, we anticipated shared signals for traits related to environmental conditions in the bobcat and Iberian lynx—both inhabiting temperate southern regions—and in the Eurasian and Canada lynx—distributed in colder northern regions. We also expected overlap in traits related to diet and habitat, particularly when comparing opportunistic species (bobcat, Eurasian lynx) to more specialist species (Iberian lynx, Canada lynx). However, our analysis did not support these expectations, probably because the axes of comparison were partly conflicting. Species occupying similar habitats differ markedly in body size and diet, whereas species with comparable ecological strategies inhabit contrasting environments. These conditions inherently limit the likelihood of detecting shared adaptive responses. Moreover, the lack of shared signatures could partly be explained by population differentiation within species, where locally adaptive variation across environments becomes masked when selection is evaluated at the species-wide level (Reding et al. 2012; Ratkiewicz et al. 2014; Rueness et al. 2014; Broderick 2020).

Nevertheless, our results revealed a significant overrepresentation of GO terms associated with neurological functions across all lynx species, although the specific terms varied among species. Felids rely heavily on strong neuro-muscular coordination, acute sensory perception, and precise timing for successful hunting (Hunter 2015), making neurological adaptations essential for their ecological and behavioral success. The *DCC* gene, associated with neuronal development and axon guidance, is a selection candidate shared by the Canada lynx and Eurasian lynx, possibly reflecting a soft sweep acting on shared ancestral variation given their sister-taxon relationship and underscoring the potential importance of neural adaptations in these species. Evolutionary pressures on neural development and related processes might have played a crucial role in shaping the unique phenotypes, behaviors, and dietary patterns observed in lynx species, as suggested by previous studies that identified selection on neuronal functions in tiger and Eurasian lynx populations (Mittal et al. 2020; Bazzicalupo et al. 2023).

### Caveats of Sweep Inference Under Complex Demographies

A factor that may hinder proper understanding of adaptative mechanisms is population demography, which has followed distinct patterns in the four extant lynx species, resulting in contrasting levels of genetic diversity, as reflected in the contrasting SNP densities observed in this study. Bobcat exhibit the largest census and effective population sizes (N_e_), concordant with its highest SNP density and the high diversity reported in previous studies (Reding et al. 2012; Janecka et al. 2016; Broderick 2020). By contrast, Iberian lynx maintained small effective population sizes throughout its history and underwent serial contractions culminating in a 20th-century nadir of <100 individuals, resulting in one of the most genetically eroded mammalian genomes described to date (Abascal et al. 2016). Between these two extremes, the Eurasian lynx is widespread across Eurasia, although its populations have been declining during the Holocene, with intense anthropic pressures resulting in the extirpation of most of its historical range in Western Europe, leaving a few genetically depauperated and differentiated populations and an overall low genetic diversity (Rueness et al. 2003, 2014; Schmidt et al. 2009; Ratkiewicz et al. 2014; Lucena-Perez et al. 2020; Bazzicalupo et al. 2022). On the other hand, the Canada lynx showed unexpectedly low SNP densities given its wide distribution and large census sizes across North America. A very low genome-wide heterozygosity, similar to that of Iberian lynx, was recently reported in the Canada lynx (Meeus et al. 2025). This could be related to its quasi-periodic demographic oscillations driven by the ten-year snowshoe-hare cycle (Elton and Nicholson 1942; Moran 1953; O’Donoghue et al. 1997; Stenseth 2004).

These demographic contrasts frame the search for genomic footprints of selection. While adaptive processes generally affect variation locally in the genome, neutral processes such as demographic history influence overall levels of genome diversity. However, demographic processes, such as recent severe bottlenecks, not only alter mean diversity, but also influence higher-order moments of diversity and linkage disequilibrium, thereby potentially confounding selection signals (Barton 1998; Jensen et al. 2005; Pavlidis et al. 2008). Besides, because the efficiency of selection lies on its ability to overcome drift, contractions in N_e_ tip the balance toward drift, permitting only alleles of large fitness effect to be maintained (Allendorf 1986; Masel 2011). Consequently, species that have endured marked bottlenecks or cyclical declines are expected to harbor fewer and more idiosyncratic selective-sweep tracts, which may also be harder to detect, than their large-N_e_ counterparts.

To address these challenges, we applied a method shown to retain high power even under recent bottlenecks (DeGiorgio and Szpiech 2022). However, its power declines steeply for older or softer sweeps (time of selection > 1,500 generations; selection coefficient < 0.01). Moreover, population structure can both create sweep-like patterns—via allele-frequency differences that mimic the rapid rise of a beneficial allele—and mask genuine sweeps if the selected haplotype is diluted across subpopulations. Consequently, the signals we report should be interpreted with caution, as some may reflect demographic artifacts. Future work that couples phasing data (e.g. using pedigree or long-read information) with joint demographic-selection inference, or that applies methods robust to population structure will therefore be essential to disentangle true adaptive events from demographic artifacts and to place our findings in a more nuanced evolutionary context.

## Conclusions

This genome-wide survey of selection across the four extant lynx species indicates that recent, strong selective events have shaped their genomes. Due to divergent ecologies and environmental pressures, each species exhibits a distinct pattern of recent adaptation. In particular, the Canada lynx shows signals related to sensory perception and pigmentation; the Eurasian lynx to cardiovascular function; the Iberian lynx to immune resistance and the bobcat to reproduction and metabolic processes, in concordance to ecological, biological and demographic challenges specific to each species.

By integrating genome scans with functional enrichment analyses, this study provides a comprehensive comparative genomic insight into adaptation in a rapidly radiating genus. The limited evidence for parallel evolution underscores the diversity of molecular routes to similar ecological outcomes. Together, these results offer a valuable framework for future investigations into the genomic basis of ecological adaptation, the interplay between selection and demography, and the molecular evolution of traits critical to survival across divergent habitats.

## Methods

### Sampling, DNA Extraction, Sequencing and Read Alignment

Whole genome sequences from a total of 80 individuals were analyzed for this study, comprising: 19 *L. canadensis*, 18 *L. rufus*, 32 *L. lynx* and 11 *L. pardinus* individuals sampled across each species’ distributional range (Table S1, Fig. 1).

Samples consisted of blood, muscle, tissue, skin, claw, and hair and were obtained either from zoos, museum collections, biologists, hunters, or state wildlife agencies under the corresponding permits. No animals were harmed during live-trapping and handling. DNA was extracted from bobcat and Canada lynx samples using the Qiagen DNeasy kit and both Eurasian and Iberian lynx samples were processed by overnight digestion using proteinase K and extracted using silica-coated paramagnetic beads (NucleoMag® Tissue, MACHEREY-NAGEL GmbH & Co. KG). Samples yielding DNA concentration too low to be sequenced were re-extracted using a classical phenol-chloroform protocol. DNA was sent to sequencing facilities for Illumina paired-end 2 × 150 bp library preparation and sequencing. Further details on samples, sequencing facilities and sequencing technology are provided in Table S1.

We performed a quality control of the raw sequenced reads using fastqc (https://www.bioinformatics.babraham.ac.uk/projects/fastqc). Adapters were removed with Trimmomatic (Bolger et al. 2014). Sequencing reads were aligned to the 2.5 Gb *Felis catus* reference genome v9.0 (GCA_000181335.4) (Buckley et al. 2020) using bwa-mem (Li 2013). After alignment, duplicate reads were marked with the MarkDuplicates function of picard (http://broadinstitute.github.io/picard), and INDELs were realigned using the GATK v3.7 (McKenna et al. 2010) commands RealignerTargetCreator and IndelRealigner. Coverage, mapping statistics and read-length distributions were calculated using samtools (Li et al. 2009).

### Variant Calling and Filtering

Variant calling was performed using GATK v.4.1.4.1 (McKenna et al. 2010). Genome-VCF (gVCF) were generated for each sample using the HaplotypeCaller command and then combined using CombineGVCFs. Using the command GenotypeGVCFs, we conjunctly called the initial set of unfiltered variants from the combined gVCF of all samples of the four species, which included a total of 95 million variant sites. The resulting VCF was filtered, removing variants found in low-mappability and repetitive regions, as well as indels, non-biallelic sites and *Lynx* genus exclusive substitutions. Low-quality variants were also filtered out following GATK's suggested thresholds of variant quality (i.e. QUAL ≤ 30, QD ≤ 2.0, FS ≥ 60.0, MQ ≤ 40.0), after inspecting the genome-wide distribution of values of these statistics. In order to exclude regions where multiple paralogs are collapsed in the reference genome, we removed variants with a read depth exceeding the average read depth plus 1.5 times the standard deviation, calculated using SAMtools depth (Li et al. 2009). Genotypes with an average depth of coverage lower than 3× per sample were also filtered out. Finally, the joint VCF was split by species using GATK SelectVariants, and each species-specific VCFs were filtered by removing sites with genotypes missing in more than 70% of samples or heterozygous in more than 80% of samples, as well as invariant sites within each species.

### Selection Scans and Candidate Regions

We explored per species signatures of positive selection using the composite likelihood ratio test for the saltiLASSI method implemented in the lassip software (DeGiorgio and Szpiech 2022). This test has high power to detect both hard and soft sweeps from positive selection of recent to moderate age by evaluating deviations in the distribution of haplotype multilocus Genotype Frequency Spectra (GFS) across the genome. Because reliable haplotype inference requires large reference panels, the authors recommend using the GFS framework when only unphased genotype data are available. Genome-wide GFS truncated at K = 10 distinct genotypes (−k) were first computed for sliding windows of 101 SNPs (−winsize) with a step size of 50 SNPs (−winstep). These spectra were used to estimate the genome-wide neutral distribution of multilocus genotypes (−calc-spec), which was subsequently used as the null model (−spectra), when evaluating deviations of each window's GFS using the saltiLASSI (−salti) statistic. At each test location (center of each window), saltiLASSI computes a log composite likelihood ratio test statistic (Λ) measuring the support for positive selection. Finally, candidate regions for positive selection were defined as the 1% of the windows with the highest likelihood value Λ. Subsequently, overlapping outlier windows—arising from the winstep set at 50—were merged into candidate regions. Because windows are defined by a fixed number of SNPs rather than a fixed physical length, they may span different genomic distances across species depending on SNP density; however, each window always contains the same number of polymorphic sites used to estimate the genotype frequency spectrum underlying the saltiLASSI statistic.

### Functional Enrichment and Protein Interaction Analysis

Genes overlapping with candidate genomic regions under selection—including an extension of 20 kbp upstream and downstream from each region—were identified based on annotations from the *Felis catus* reference genome v9.0 (GCA_000181335.4) using Ensembl IDs. By extracting genes, we transitioned from genomic loci of interest to candidate genes harboring signatures of positive selection.

To test for statistical overrepresentation of gene functions, a functional enrichment analysis was conducted with the species’ candidate genes using the R package topGO (Alexa and Rahnenfuhrer 2017), with gene classification based on Gene Ontology (GO) term annotations (Ashburner et al. 2000; The Gene Ontology Consortium et al. 2023). Overrepresentation tests were conducted by comparing the frequency of each GO term within the Biological Process (BP) ontology in the candidate gene set to its frequency in the background gene universe. This background consists in all genes annotated in the Felis catus reference genome with at least one GO term annotation. Enrichment was assessed using Fisher's exact test combined with the weight01 algorithm, which accounts for the hierarchical dependency structure of the GO graph by iteratively down-weighting genes contributing to significant child terms when testing related parent terms. Only significant *P*-values (Fisher's exact test, *P* < 0.05) are reported.

Protein–protein interaction networks were analyzed using the STRING database (Szklarczyk et al. 2023). Lists of candidate genes were uploaded using the multiple proteins option and mapped to the *Felis catus* reference proteome. Interaction networks and statistics were constructed using default parameters. The significance of network connectivity was evaluated using the STRING protein–protein interaction (PPI) enrichment test, which compares the observed number of interactions with the number expected for a random set of proteins of similar size. Functional modules within the networks were identified using STRING clustering, and enrichment of biological processes within clusters was assessed using Fisher's exact test with false discovery rate (FDR) correction.

## Supplementary Material

evag086_Supplementary_Data

## Data Availability

Sequences analyzed in this study are available on the European Nucleotide Archive under the primary accession numbers: PRJEB109976 (current study), PRJEB48088 (Bazzicalupo et al. 2022), PRJEB28038 (Lucena-Perez et al. 2020) and PRJEB12609 (Abascal et al. 2016). Scripts used for bioinformatics analyses are available in: https://github.com/lorenalorenzo/selection_scan_lynx
